# Bumble bee parasite strains vary in resistance to phytochemicals

**DOI:** 10.1038/srep37087

**Published:** 2016-11-24

**Authors:** Evan C. Palmer-Young, Ben M. Sadd, Philip C. Stevenson, Rebecca E. Irwin, Lynn S. Adler

**Affiliations:** 1 Department of Biology, University of Massachusetts at Amherst, Amherst, Massachusetts 01003, United States; 2 School of Biological Sciences, Illinois State University, Normal, Illinois 61790, United States; 3 Royal Botanic Gardens, Kew, Richmond, Surrey TW9 3AE, United Kingdom; 4 University of Greenwich, Medway, ME4 4TB, United Kingdom; 5 Department of Applied Ecology, North Carolina State University, Raleigh, North Carolina 27695, United States

## Abstract

Nectar and pollen contain diverse phytochemicals that can reduce disease in pollinators. However, prior studies showed variable effects of nectar chemicals on infection, which could reflect variable phytochemical resistance among parasite strains. Inter-strain variation in resistance could influence evolutionary interactions between plants, pollinators, and pollinator disease, but testing direct effects of phytochemicals on parasites requires elimination of variation between bees. Using cell cultures of the bumble bee parasite *Crithidia bombi*, we determined (1) growth-inhibiting effects of nine floral phytochemicals and (2) variation in phytochemical resistance among four parasite strains. *C. bombi* growth was unaffected by naturally occurring concentrations of the known antitrypanosomal phenolics gallic acid, caffeic acid, and chlorogenic acid. However, *C. bombi* growth was inhibited by anabasine, eugenol, and thymol. Strains varied >3-fold in phytochemical resistance, suggesting that selection for phytochemical resistance could drive parasite evolution. Inhibitory concentrations of thymol (4.53–22.2 ppm) were similar to concentrations in *Thymus vulgaris* nectar (mean 5.2 ppm). Exposure of *C. bombi* to naturally occurring levels of phytochemicals—either within bees or during parasite transmission via flowers—could influence infection in nature. Flowers that produce antiparasitic phytochemicals, including thymol, could potentially reduce infection in *Bombus* populations, thereby counteracting a possible contributor to pollinator decline.

Flowers can act as intermediaries for the transmission of plant and animal diseases^1^. These diseases include infections of economically and ecologically important pollinators, many species of which are threatened by decline related to the interaction of several factors, including parasites^2^,^3^,^4^. For example, honey bee viruses have been found on pollen grains^5^,^6^, and bumble bee and honey bee parasites, including the internationally distributed *Nosema* spp. and *Crithidia* spp., can be spread between bee colonies and species that forage on the same plants^7^. This transmission can have devastating consequences for native pollinator populations^8^,^9^.

While flowers can act as sites of parasite transfer^10^, they also provide food for pollinators. Bee diets consist of floral nectar and pollen that provide carbohydrates and proteins for bee growth and development^11^. In addition to macronutrients, floral rewards also contain phytochemicals^12^,^13^, including the major secondary compound classes alkaloids, phenolics, and terpenoids^14^. Floral phytochemicals may have a variety of ecological functions, including acting as antimicrobial agents in both plants and the animals that consume them^1^. For example, (*E*)-β-caryophyllene can protect pollen and floral tissue from infection by plant pathogens^15^. Likewise, animals that consume antimicrobial phytochemicals may gain protection from their own parasites, as shown in herbivores^16^,^17^,^18^. In pollinators, ingestion of floral phytochemicals^19^ and certain types of honey^20^ were therapeutic for infected honey bees (*Apis mellifera*). Infection also stimulated collection of phytochemical-rich resins^21^ and preference for high-phytochemical nectar^22^,^23^, indicating the potential for phytochemicals to improve pollinator health.

Many phytochemicals found in flowers have direct activity against trypanosomes^24^,^25^. For example, gallic acid was lethal to *Leishmania donovani*^26^, and thymol and eugenol inhibited growth of *Trypanosoma cruzi* and *Crithidia fasciculata*^27^. It is therefore likely that some floral phytochemicals may inhibit trypanosome parasites of bumble bees. *Crithidia bombi*^28^ is an intestinal trypanosome parasite of bumble bees (*Bombus* spp.) that decreases queen survival and colony fitness^29^ and may exacerbate the negative effects of pesticides^30^ and nutritional stress^31^. *Crithidia bombi* encounters phytochemicals throughout its life cycle, making it a relevant system for testing the effects of phytochemicals on pollinator infection^22^,^23^,^32^,^33^. Parasites infect new hosts via transmission at flowers^10^ and within bee hives^32^, which contain derivatives of nectar, pollen, and other plant materials^21^. *Crithidia bombi* has not been detected in floral nectar^34^. However, within hosts, *C. bombi* inhabits the gut lumen, where cells have direct exposure to host-ingested nectar and pollen phytochemicals in the crop, and possibly also in the mid- and hindgut. In contrast to trypanosomes that infect the circulatory system or organs of their hosts, intestinal *C. bombi* lacks a physical barrier to shield it from ingested compounds, and may be exposed to phytochemical concentrations that approach those found in nectar and pollen. Hence, oral consumption of phytochemicals by bees could have strong and direct effects on parasites, and the phytochemical concentration that inhibits parasite growth *in vitro* may provide an estimate of the oral dose that could ameliorate infection in hosts.

Several studies have demonstrated that phytochemical ingestion by *B. impatiens* and *B. terrestris* reduces *C. bombi* infection. Five phytochemicals found in nectar—gelsemine^33^, nicotine^22^,^23^, anabasine, thymol, and catalpol^22^ – reduced *C. bombi* infection intensities. However, both the magnitude and direction of effects of phytochemicals on *C. bombi* varied among studies. For example, other studies found that thymol^35^ and anabasine^36^ did not affect *C. bombi* infection, and nicotine increased infection intensity^36^. Taken together, these results suggest that phytochemicals have variable effects on *C. bombi* infection, with effects dependent on the unique combination of parasite strain, host genotype, and abiotic conditions used in each experiment. Therefore, an approach that eliminates host-related variability would help to determine the direct effects of phytochemicals on parasites, and allow comparisons of phytochemical sensitivity among parasite strains.

Both *C. bombi* strains and floral phytochemical concentrations are variable. *Crithidia bombi* populations are genetically^37^ and phenotypically diverse^32^. Inter-strain variation could determine resistance to phytochemicals—defined here as the ability to survive, grow, and reproduce when exposed phytochemicals—as has been demonstrated within populations of other pathogenic microbes, such as quinine- and artemisinin-resistant *Plasmodium falciparum*^38^. Like parasite strains, floral phytochemical concentrations are variable, and have dose-dependent effects on both pathogens and hosts^39^. For example, nectar nicotine and anabasine concentrations spanned multiple orders of magnitude among related *Nicotiana* species^40^. Within a species, nectar nicotine varied between *Nicotiana attenuata* plant populations, within populations, and across a six-fold range between flowers of a single inflorescence^41^. Similarly, nectar concentrations of *Rhododendron ponticum* grayanotoxins varied between native and invasive populations and within patches^42^. Testing a range of parasite strains, phytochemicals and concentrations in a single study could identify candidate medicinal compounds and illustrate the potential effects of phytochemicals on pollinator parasites in nature.

We used a standardized, high-throughput protocol to test the direct effects of different phytochemicals against multiple parasite strains across a range of chemical concentrations. Cell culture-based methods have been used to quantify the effects of phytochemicals on insect-vectored trypanosome species such as *Leishmania donovani*^24^, *Trypanosoma cruzi*^27^,^43^, and *Trypanosoma brucei*^24^,^44^,^45^ that cause disease in humans and are close phylogenetic relatives of *C. bombi*^46^. Here, we extend a previously described *C. bombi* cell culturing method^47^ to assess variation in the direct effects of nine floral phytochemicals—two alkaloids, one cyanogenic glycoside, a hydroxycinnamic acid, a hydroxycinnamic acid ester, a gallic acid, a phenylpropene, and two terpenoids—on four different *C. bombi* strains. We also searched published literature to compare phytochemical sensitivity of *C. bombi* to that of other trypanosome species, animal cells, and insects. To gauge the ecological relevance of each phytochemical’s effects in culture, we combined field sampling of five plant species with literature searches to quantify phytochemical concentrations in nectar and pollen.

## Results

### Cell culture experiments

**Figure 1:**
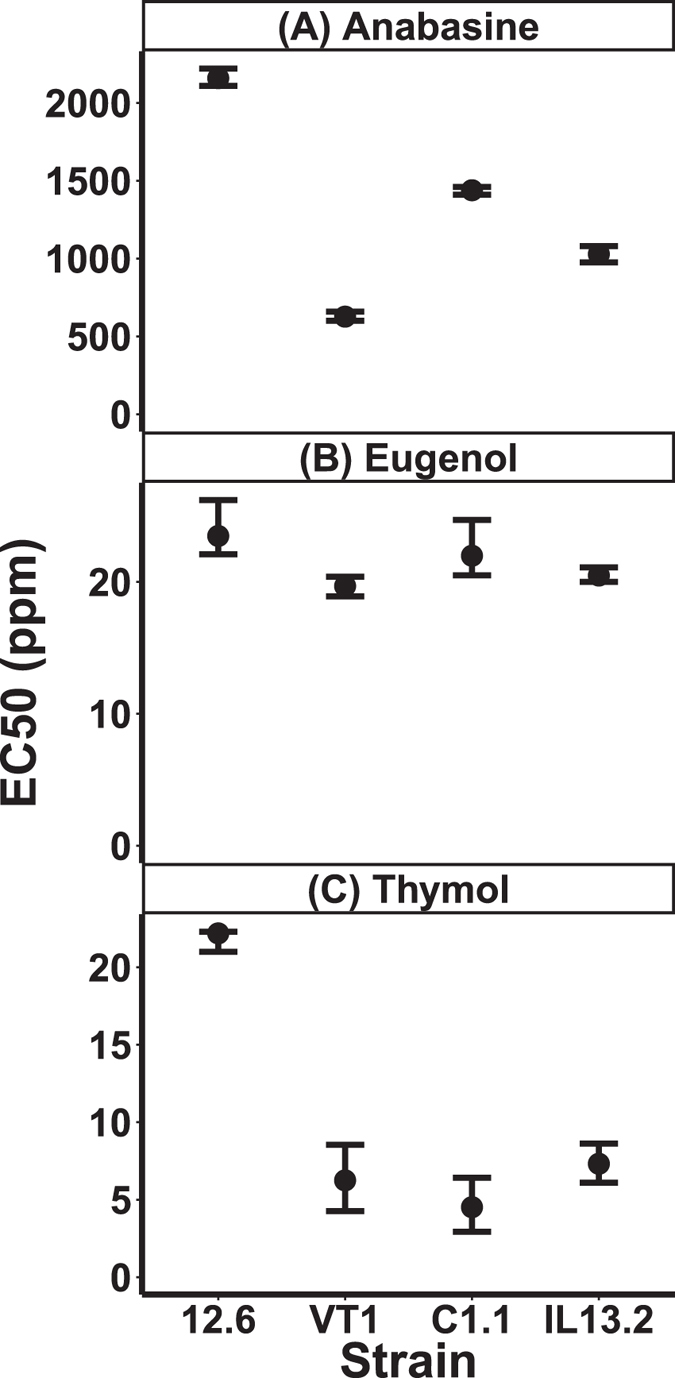
Inhibitory effects of (A) anabasine, (B) eugenol, and (C) thymol against 4 strains of *C. bombi.* Points indicate EC50 values in ppm phytochemical. Error bars show 95% credible intervals derived from Bayesian Markov Chain Monte Carlo model fit (see Materials and Methods). For each strain (x axis) and phytochemical (vertically arranged panels), model fit was derived from growth on a 96-well plate at 6 phytochemical concentrations (n = 8 (anabasine), 6 (eugenol), or 7 (thymol) replicate samples per concentration). See Supplementary Figures S1–S3 for complete dose-response curves and confidence bands from the fitted models, and Supplementary Figure S4 for representative growth curves of OD over time.

In comparison to other trypanosome species, *C. bombi* were remarkably resistant to common phytochemicals, with no growth inhibition at concentrations previously found to lower infection intensity in nectar fed to live bees (Table 1). Among the alkaloids, nicotine at doses of up to 1000 ppm had no effect on growth, and over 1000 ppm anabasine was required for 50% growth inhibition (EC50, Table 1, Fig. 1, Supplementary Figure S1). None of the tested strains were susceptible to the cyanogenic glycoside, amygdalin, nor to the antitrypanosomal phenolics caffeic acid, chlorogenic acid, and gallic acid, even at concentrations that were several orders of magnitude above the inhibitory thresholds of related pathogens (Table 1). The sesquiterpene β-caryophyllene also did not inhibit growth of any strain at concentrations up to 50 ppb. Of the nine phytochemicals tested, only three—anabasine, eugenol, and thymol—were sufficiently inhibitory to estimate dose-response curves and EC50 values (Fig. 1, Table 1, Supplementary Figures S1–S3).

Strains varied in resistance to all three inhibitory compounds. Significant variation was found in resistance to anabasine (Fig. 1A). Each strain exhibited a distinct level of resistance, which varied among strains by more than 1500 ppm. The most sensitive strain, VT1 (EC50 = 628 ppm, 95% Bayesian Credible Interval (CI): 601–659 ppm), was inhibited by one-third the anabasine concentration of the most resistant strain, 12.6 (EC50 = 2160 ppm, 95% CI: 2110–2220 ppm). The other two strains, IL13.2 (EC50 = 1030 ppm, 95% CI: 975–1080 ppm) and C1.1 (EC50 = 1440 ppm, 95% CI: 1410–1440 ppm), were intermediate in resistance.

Eugenol resistance (Fig. 1B) was the most consistent across strains, with all EC50 values between 19.7 and 23.5 ppm, yet the non-overlapping 95% credible intervals (CI) still indicated statistically significant variation. The relative resistance ranks of the four strains were the same as for anabasine and eugenol: Strain VT1 (EC50 = 19.7 ppm, 95% CI: 18.9–20.4 ppm) was again the most sensitive, and strain 12.6 the most resistant (EC50 = 23.5 ppm, 95% CI: 22.1–26.2 ppm); intermediate resistance was observed in IL13.2 (EC50 = 20.5 ppm, 95% CI: 20.0–21.1 ppm) and C1.1 (EC50 = 22.0 ppm, 95% CI: 20.5–24.7 ppm).

Resistance to thymol (Fig. 1C) was also variable. As was the case for the other two compounds, strain 12.6 (EC50 = 22.2 ppm, 95% CI: 22.3–21.0 ppm) was the most resistant, with more than three times the resistance of the other three strains, which were not significantly different from one another (VT1, EC50 = 6.26 ppm, 95% CI: 4.27–8.55 ppm; C1.1, EC50 = 4.53 ppm, 95% CI: 2.93–6.42 ppm; IL13.2, EC50 = 7.33 ppm, 95% CI: 6.10–8.62 ppm).

### Naturally occurring phytochemical concentrations

Using published literature and field sampling, we surveyed ecologically relevant pollen, nectar, and honey concentrations of the nine phytochemicals tested against *C. bombi* (Table 2). In comparison to published values for honey, our own analyses indicated very high levels of chlorogenic acids in the pollen of the crop species *Persea americana* (avocado), *Malus domestica* (apple), and *Vaccinium corymbosum* (blueberry, both wild and cultivated; Table 2). In the three plant taxa for which we analyzed both pollen and nectar, concentrations of the chlorogenic acid 5-caffeoylquinic acid were 25- to 30-fold higher in pollen than in nectar (Wilcoxon W-test, *M. domestica*: W = 25, *P* < 0.001; *V. corymbosum* (cultivated): W = 18, *P* < 0.001; *V. corymbosum* (wild): W = 0, P < 0.001). Although nectar chlorogenic acid concentrations were lower than pollen concentrations, nectar concentrations were still several orders of magnitude higher than those recorded in honey, with the exception of *Leptospermum scoparium* honey (Table 2). Similarly, thymol concentrations in the nectar of *Thymus vulgaris* were over 10-fold above the highest value recorded for natural honey (Table 2), despite air-drying of samples prior to measurement (see Materials and Methods).

## Discussion

*Crithidia bombi* was far less susceptible to the tested trihydroxybenzoic and hydroxycinnamic phenolic phytochemicals than were other, previously studied bloodstream trypanosomes. *L. donovani* and *T. brucei*, for example, were inhibited by <10 ppm of gallic acid^26^,^48^, whereas concentrations up to 250 ppm had minimal effects on any tested strains of *C. bombi*. Similarly, caffeic acid, which inhibited *L. donovani* and *T. brucei* at <10 ppm^24^, had no effect on *C. bombi* strains at concentrations up to 250 ppm. Furthermore, the EC50 for chlorogenic acid against *C. bombi* was >2500 ppm, which was 100 times higher than the EC50 for *L. donovani* (EC50 7–17 ppm^49^,^50^) and *T. brucei* (18.9 ppm^49^). Although some variation in EC50 estimates could reflect methodological differences between our study and previous investigations, a difference of such magnitude for multiple phytochemicals provides strong evidence of comparatively high phytochemical resistance in *C. bombi*. This exceptional level of resistance may reflect the evolutionary history of *C. bombi*. In contrast to *L. donovani* and *T. brucei*, which are transmitted by blood-feeding insects and would be expected to have comparatively little direct exposure to phytochemicals, *C. bombi* may be adapted to chronic phytochemical exposure in the intestine of nectar- and pollen-consuming bumble bees. Bumble bees are generalist pollinators that consume nectar and pollen from a wide range of plant species^11^. Both nectar^51^ and pollen^14^ contain diverse compound mixtures, to which *C. bombi* in the gut lumen would be directly exposed^52^, particularly in the proximal parts of the gut, before phytochemicals are absorbed or metabolized by hosts or commensalists. Study of the mechanisms by which *C. bombi* withstands such high phytochemical concentrations could offer insight into the evolution of chemical resistance in medically important trypanosomes.

In addition to being less susceptible to phytochemicals than were other trypanosomes, *C. bombi* showed no growth inhibition at phytochemical concentrations exceeding those documented in honey (Table 1, Table 2). For example, for the known antitrypanosomal compound caffeic acid, *C. bombi* was not inhibited by 250 ppm (Table 1), over 9 times the maximum honey value of 26.8 ppm (Table 2, range 0.76–26.8 ppm for 14 honey types)^53^; for gallic acid, *C. bombi* was again robust to 250 ppm (Table 1), or 3 times the maximum reported honey value of 82.5 ppm (Table 2; among 14 honey types, only oak honey exceeded 1 ppm gallic acid)^53^.

There are a number of nonexclusive explanations for the insensitivity of *C. bombi* to phytochemicals above their natural concentration range. First, the phytochemical concentrations found in honey samples may underestimate naturally occurring concentrations. Fanning of nectar to produce honey^11^, as well as prolonged storage, may evaporate volatile nectar components such as thymol, eugenol, and β-caryophyllene and could promote oxidation of phenolic compounds^54^. The thymol and chlorogenic acid concentrations measured in our field samples (Table 1), which were orders of magnitude higher than the values for honey found in the literature, illustrate this point. Second, in natural settings, phytochemicals are encountered in complex combinations, such that total phytochemical concentrations of biologically active compounds may far exceed the concentration of any one chemical component. Pollen comprises a mixture of phytochemicals, with the sum concentration of all phenolic constituents reaching 1.3–8.2% phenolics by weight (13,000–82,000 ppm)^55^. Even honey may contain up to 12,000 ppm total phenolics (range 1,600–12,000 ppm)^53^. Third, in their hosts, parasites are subject to additional antimicrobial chemicals produced by the host immune system and competing gut microbiota. Multiple antimicrobial peptides produced by bees have synergistic effects with one another^56^, and should be tested for synergy with floral phytochemicals as well. The *Bombus* gut microbiome includes species that produce ethanol and organic acids^57^, which also inhibit microbial growth^58^,^59^. Hence, the high resistance of *C. bombi* that we observed to single phytochemicals may be necessary to tolerate the effects of multiple phytochemicals, antimicrobial peptides, and microbiome-derived toxins acting in concert. Future experiments should explicitly address the interactive effects of multiple phytochemicals in combination.

In addition to explaining why *C. bombi* has such high resistance to individual phytochemicals under optimal conditions, the interactive effects of multiple factors may explain why low concentrations of phytochemicals were sufficient to decrease parasitism in live bees^22^. All tested strains of *C. bombi* were resistant to phytochemicals at concentrations 100 times higher than those previously shown to be medicinal in *B. impatiens* and *B. terrestris*. Our strains were not inhibited by up to 1000 ppm nicotine, or 500 times the 2 ppm previously found to ameliorate infection in bees^22^,^23^. Our lowest EC50 value for anabasine (628 ppm) was still over 100-fold higher than the 5 ppm previously shown to reduce infection levels^22^. Inhibitory concentrations of thymol, where the minimum EC50 of the four strains was 4.5 ppm, were likewise more than 20-fold the 0.2 ppm medicinal concentration in *B. impatiens*^22^. These discrepancies far exceed the ~3-fold variation found among strains in our study, indicating that differences between *in vitro* and *in vivo* inhibitory concentrations do not merely reflect the use of different strains in our study versus previous live-bee experiments. We suggest that the low phytochemical concentrations necessary to ameliorate host infection may reflect phytochemical-induced changes in hosts, which could complement the direct effects of phytochemicals on parasites. For example, phytochemical ingestion may act indirectly on parasites by modulating the host immune response, as shown in humans^60^ and in honey bees, where a honey constituent increased expression of genes that encode antimicrobial peptides^61^. Phytochemicals could also act as antioxidants that scavenge free radicals^62^ and reduce the deleterious effects of pathogens^39^. Studies of live bees are needed to define how phytochemicals exert indirect effects on parasite infection via modulation of host immunity or behavior, such as induction of antimicrobial peptides or stimulation of intestinal motility that expels parasites from the gut^63^.

Our four *C. bombi* strains varied in resistance to the three phytochemicals that inhibited growth, spanning a five-fold range for thymol and a three-fold range for anabasine. Overall, strain “12.6” exhibited both the fastest growth (Supplementary Figures S1–S4) and the highest phytochemical resistance (Fig. 1). Strains with a high rate of growth might be able to form biofilms that provide protection from growth-inhibiting chemicals, or metabolize the chemicals before deleterious effects are realized. Studies that use a greater number of strains are needed to test for positive correlations between phytochemical resistance and growth rate, both in cell cultures and in live bees, where *C. bombi* exists within a diverse microbial community^64^. Alternatively, negative correlations could reflect trade-offs between resistance and growth or infectivity. Variation in phytochemical resistance among parasites could be a target and possibly a result of natural selection. At the landscape scale, regional parasite and plant sampling, combined with cell culture experiments, could establish whether parasites show evidence of adaptation to phytochemicals characteristic of their local plant community. These correlative studies could be complemented by experiments that test how parasites respond to chronic phytochemical exposure, and whether resistance can evolve over time.

Our sampling data show that thymol inhibited *C. bombi* at concentrations found in *T. vulgaris* nectar. The range of EC50 values for *C. bombi* (4.5 to 22 ppm) spanned the natural range of thymol concentrations in *T. vulgaris* nectar (5.2–8.2 ppm). Although nectar concentrations did not completely inhibit growth, 50% growth inhibition could meaningfully decrease the intensity of infection and its negative effects on bees. Also, because it is likely that some thymol was lost during sample processing, our measurements may provide a conservative estimate of thymol-mediated inhibition by *Thymus* nectar. Thymol is used prophylactically to combat *Varroa* mite infestations^65^, and inhibited *Nosema* infection in *A. mellifera*^19^ and *Crithidia* infection in *B. impatiens*^22^. Although it is possible that nectar thymol is absorbed or metabolized by bees or their gut commensalists, or diluted through combination with nectar of other species, phytochemicals are detectable in the lumen post-ingestion^52^, and even very low nectar concentrations (0.2 ppm) can reduce *C. bombi* infection intensity in *B. impatiens*^22^. Because individual bumble bees generally forage from only one or several floral species^66^, consumption of medicinally relevant amounts of thymol would seem plausible in the wild. Our study builds on prior results by reporting concentrations of thymol in floral nectar for the first time, and documenting the direct activity of this phytochemical against multiple parasite strains at naturally occurring concentrations.

Thymol and eugenol have been shown to possess broad-spectrum antimicrobial activity against bacteria^39^, fungi^67^,^68^, and trypanosomes^25^. These hydrophobic compounds readily penetrate and disrupt cell and mitochondrial membranes, thereby disrupting ionic gradients and causing leakage of reactive oxygen species^69^. Reactive oxygen species can oxidize monoterpenes and phenylpropenes like thymol and eugenol, which both contain double bonds and free hydroxyl groups. Oxidized phytochemicals can then initiate a free radical cascade that damages cell lipids and proteins^69^, leading to disruptions of organelle function and energy production in trypanosomes^25^. Rapidly dividing cells are especially susceptible, because they are easily penetrated during cell division^69^. Although high phytochemical concentrations are toxic to animal intestinal cells as well as to microbes, with 25 ppm thymol and 80 ppm eugenol inducing apoptosis and necrosis within 24 h^39^, the intestinal cells with direct phytochemical exposure may provide a renewable barrier between the gut lumen and the systemic circulation of multicellular animal hosts.

Phytochemicals such as thymol and eugenol, which display strong antimicrobial activity but are relatively benign to bees^70^, could have high medicinal value for both wild and managed bees that have access to plants containing these compounds. In general, bees are less susceptible than are microbes to toxic effects of essential oils^70^, and can be attracted to relevant antimicrobial concentrations^71^, which would increase the likelihood of voluntarily ingesting medicinally significant amounts of these phytochemicals under natural conditions. Eugenol, which has been found in over 400 plant species from 80 families^72^, has been shown to stimulate bee foraging and pollen collection in bumble bees^73^; 50 ppm eugenol in sugar water was attractive to honey bees^74^, whereas only 19.7–23.5 ppm inhibited *C. bombi* growth in our study. Similarly, the *A. mellifera* 14-day LD50 for thymol exceeded 1000 ppm^70^, far higher than the 4.5–22.3 ppm thymol that inhibited our *C. bombi*. Future studies should test whether availability of flowers containing thymol (such as *T. vulgaris*) or eugenol is sufficient to reduce bee parasitism in the field; such plant species could be recommended to gardeners and as hedgerow species in agricultural areas. Additional studies that examine correlations between plot- and landscape-level plant species composition and pollinator parasite loads will yield additional ecological insights.

Our field sampling revealed higher levels of phytochemicals in nectar and pollen compared to previous reports of the same phytochemicals in honey. For example, the 5.2–8.2 ppm nectar thymol measured in this study is more than ten times greater than the highest reported concentration in natural honey (Table 2). For chlorogenic acid, we identified three species with pollen concentrations >400 ppm, which is 50 times the highest value previously reported for honey (Table 2). Our findings highlight large differences between the phytochemical composition of nectar and honey, and indicate the need for more comprehensive sampling of nectar and pollen, including volatile compounds such as eugenol, to establish the types and concentrations of phytochemicals to which parasites might be naturally exposed. Sampling bumble bee honey in addition to honey bee honey may also reveal differences in chemical composition due to variation in foraging preferences or post-collection processes. Future sampling efforts will identify candidate antimicrobial phytochemicals for future testing in bees and other pollinators, and also document which floral species are sources of known antiparasitic compounds. Given the relatively unexplored nature of nectar and pollen relative to leaf phytochemistry, further sampling has significant potential to uncover new compounds of ecological and potentially medical significance.

Collectively, our experiments demonstrate the ecological and evolutionary relevance of direct effects of phytochemicals on a pollinator parasite. We show that the bumble bee parasite *C. bombi* is less susceptible to phytochemicals than are bloodstream trypanosomes, is inhibited by some nectar and pollen phytochemicals at naturally occurring concentrations, and exhibits inter-strain variation in resistance. Our results emphasize the importance of inter-strain variation and concentration-dependent responses in explaining the effects of phytochemicals on pollinator diseases, and highlight the need for additional analysis of nectar and pollen to profile the full range of phytochemicals and concentrations that occur in nature.

## Methods

### Parasite culturing

Parasite strains, each derived from a single *C. bombi* cell, were isolated from wild bumble bees collected near West Haven, CT, United States in 2012 (“12.6”, from *B. impatiens,* courtesy Hauke Koch); Hanover, NH, United States in 2014 (“VT1”, from *B. impatiens,* courtesy lab of Rebecca Irwin); Corsica, France in 2012 (“C1.1”, from *B. terrestris*, collected by Ben Sadd); and Normal, IL, United States in 2013 (“IL13.2”, from *B. impatiens*, collected by Ben Sadd). Strain 12.6 was isolated by diluting homogenized intestinal tracts of infected *B. impatiens* to 1 cell μL^−1^, then adding 1 μL of the cell suspension to wells of a 96-well plate containing *Crithidia* growth medium^47^ with the addition of 2% antibiotic cocktail to combat bacterial and fungal contaminants (penicillin 6 mg mL^−1^, kanamycin 10 mg mL^−1^, fluorcytosin 5 mg mL^−1^, chloramphenicol 1 mg mL^−1^ as described^47^). The remaining strains were isolated by flow cytometry-based single cell sorting of homogenized intestinal tracts (strain VT1) or bee feces (C1.1 and IL13.2) as described previously^47^. All strains were isolated directly from wild bees with the exception of VT1, which was first used to infect laboratory colonies of *B. impatiens* (provided by Biobest, Leamington, ON, Canada). The cell used to initiate the parasite culture was obtained from an infected worker of one of the commercial colonies. Cultures were microscopically screened to identify samples with strong *Crithidia* growth and absence of bacterial or fungal contaminants, then stored at −80 °C in a 2:1 ratio of cell culture:50% glycerol until several weeks before the experiments began. Thereafter, strains were incubated at 27 °C and propagated weekly in 5 mL tissue culture flasks (300–500 μL cultured cells in 5 mL fresh culture medium)^47^.

### Phytochemicals for cell culture assays

Phytochemicals were chosen to facilitate comparison with published work assessing *C. bombi* inhibition in *B. impatiens*^22^,^36^. Additional compounds were selected based on widespread presence in flowers, nectar, honey, or pollen and documented anti-trypanosomal activities (Tables 1 and 2). We tested the effects of nine compounds: the pyridine alkaloids nicotine (Sigma-Aldrich, St. Louis, MO) and anabasine (Sigma-Aldrich), the cyanogenic glycoside amygdalin (Research Products International, Mt. Prospect, IL), the cinnamic acid caffeic acid (Indofine, Hillsborough, NJ), the cinnamic acid ester 3-caffeoylquinic acid (“chlorogenic acid”, Biosynth International, Itasca, IL), the phenylpropene eugenol (Acros, Thermo Fisher, Franklin, MA), the trihydroxybenzoic phenolic gallic acid (Acros), the sesquiterpene β-caryophyllene (SAFC, Milwaukee, WI), and the monoterpene alcohol thymol (Fisher Scientific, Franklin, MA).

Phytochemical treatment media were prepared by dissolving stock chemicals either directly in medium followed by sterile filtration (for the more soluble nicotine, anabasine, amygdalin, chlorogenic acid, and eugenol) or by pre-dissolving compounds in ethanol (for the less soluble caffeic acid, gallic acid, β-caryophyllene, thymol). Treatment concentrations were chosen to span the range of concentrations known to occur in plant nectar and pollen (Table 1) and/or inhibit trypanosomes (Table 2), with maximal concentrations limited by compound solubility. For experiments using dilutions prepared from an ethanol-based stock, we equalized the ethanol concentration in each treatment by adding ethanol (up to 1% by volume, depending on the phytochemical) to the treatments of lesser concentrations.

### Experimental design

We conducted 9 experiments, each testing all 4 parasite strains in parallel against a single phytochemical. Cell cultures (1 mL) were transferred to fresh medium (5 mL) and allowed to grow for 48 h in tissue culture flasks. Immediately before the assay, cultures were transferred to 50 mL centrifuge tubes and centrifuged for 10 min at 10,000 g. The supernatant was removed and the cells were resuspended in 3 mL fresh medium. Cell density of the resulting suspension was calculated by counting parasite cells at 400x magnification using a Neubauer hemocytometer. Each strain was adjusted to a cell density of 1,000 cells μL^−1^.

A separate 96-well plate was prepared for each strain, i.e., 4 plates per experiment, one for each of the four strains. Each plate contained eight replicate wells at each of six phytochemical concentrations, with each concentration assigned to columns 3–10 of a given row to minimize edge effects. To each well, 100 μL of 1,000 cells μL^−1^ cell suspension was added to 100 μL of the phytochemical-enriched treatment medium using a multichannel pipette, resulting in a starting cell density of 500 cells μL^−1^. The outer wells of the plate (columns 1, 2, 11, and 12, plus the remaining wells in rows A and B) were filled with 100 μL treatment medium (8 wells per concentration) and 100 μL control medium; these wells were used to control for changes in optical density (OD) unrelated to cell growth. Plates were incubated for 5 d at 27 °C on a microplate shaker (250 rpm, 3 mm orbit). OD readings (630 nm) were taken at 24 h intervals, as described previously^75^, immediately after resuspending the cells (40 s, 1000 rpm, 3 mm orbit) using the microplate shaker. We calculated net OD (i.e., the amount of OD resulting from parasite growth) by subtracting the average OD reading of cell-free control wells of the corresponding concentration, plate, and timepoint. For analysis of assays using the volatile phytochemicals eugenol and thymol, we excluded the replicates closest to the control wells that contained highest phytochemical concentrations (2 per treatment for eugenol, 3 per treatment for thymol). These replicates had markedly reduced growth compared to other samples in the same treatments; we attributed this growth reduction to exposure to phytochemicals that volatilized from the neighboring control wells.

### Statistical analysis of cell culture experiments

Dose-response curves for each strain and phytochemical were computed for the three phytochemicals for which the highest tested concentration resulted in complete inhibition of growth—near-complete inhibition is necessary for accurate estimation of the concentration that inhibits growth by 50% (EC50). All statistical analysis was carried out using the open source software R v3.2.1^76^ following methods used for antimicrobial peptides^56^. For each sample, the growth integral (i.e., area under the curve of net OD vs. time) was calculated by fitting a model-free spline to the observed OD measurements using grofit^77^. The relationship between phytochemical concentration and growth integral was modeled with a Markov chain Monte Carlo algorithm using Just Another Gibbs Sampler^78^ in combination with the R-package rjags^79^. We used the following model to describe the relationship between phytochemical concentration (*c*) and growth integral (*g*):


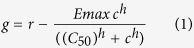


where *r* denotes growth in the absence of the phytochemical, *E*_*max*_ represents the maximum effect at high concentrations, and *C*_50_ is the phytochemical concentration at which 50% of the maximum effect is reached. The parameter *h*, the Hill coefficient, indicates how steeply the effect increases around the concentration *C*_50_. From this model, we derived parameter estimates and 95% highest posterior density credible intervals (CI) of the EC50 for each phytochemical. We defined strains as having significant differences in resistance when their 95% CI’s did not overlap. Each strain’s dose-response curve and EC50 were calculated independently of the other strains; in other words, the EC50 represents the phytochemical concentration resulting in 50% of maximal inhibition for a particular strain.

### Field sampling

#### Nectar and pollen collection

Nectar and pollen were collected from agricultural and wild species in Massachusetts and California in 2014 and 2015 (see Supplementary Table S1 for sampling locations, dates, and cultivars). We quantified thymol in *Thymus vulgaris* nectar and chlorogenic acids in *Malus domestica* (domestic apple), wild and cultivated *Vaccinium corymbosum* (blueberry), *Prunus dulcis* (almond), and *Persea americana* (avocado). Up to 10 samples of each tissue were collected, typically from each of three cultivars for agricultural species. For *Thymus vulgaris* cv. Silver, few plants were in flower at the time of collection, so it was only possible to collect enough nectar for a single nectar sample.

Pollen samples were collected using clean forceps by pinching off anthers, avoiding as much filament as possible. We collected at least 5 mg per sample, consisting of pollen, the pollen sac, and a small amount of filament. We collected from mature, undehisced or newly dehiscing anthers only. In most species, pollen was pooled across flowers within plants, but not across plants. Nectar samples were collected using separate glass microcentrifuge tubes. Care was taken to avoid contaminating samples with pollen. Depending on the plant species, we collected nectar through the corolla opening, or by removing and gently pressing the corolla to produce nectar at the flower base. Each nectar sample contained at least 5 μL but typically 20 μL nectar, added to 80 μL EtOH to prevent spoilage. Nectar was often pooled across individual plants to obtain sufficient volumes per sample. Samples were kept on ice in the field and then stored at −20 °C until lyophilization. Alcohol from *Thymus* nectar samples was evaporated at room temperature. We acknowledge that some thymol, which is volatile, may have been lost from the samples during evaporation, which we deemed necessary to prevent spoilage during shipping. As a result, our results may underestimate true nectar concentrations of this phytochemical.

#### Analysis of chlorogenic acids

Pollen samples were extracted in methanol following previously published methods^80^. Unground pollen (5–50 mg) was sonicated for 10 min with 1 mL methanol in a 2 mL microcentrifuge tube, then incubated without shaking for an additional 24 h at room temperature. Samples were centrifuged for 5 min at 12,000 rpm, and the supernatants analyzed by liquid chromatography (LC) using High Resolution Electrospray Ionisation Mass Spectroscopy (HRESIMS). Chlorogenic acids were identified based on spectral comparisons with authentic standards in the library at Royal Botanic Gardens, Kew, UK. HRESIMS data were recorded using a Thermo LTQ-Orbitrap XL mass spectrometer coupled to a Thermo Accela LC system performing chromatographic separation of 5 μl injections on a Phenomenex Luna C18(2) column (150 mm × 3.0 mm i.d., 3 μm particle size) with a linear mobile phase gradient of 10–100% aqueous MeOH containing 0.1% formic acid over 20 min. The column temperature was maintained at 30 °C with a flow rate of 0.5 ml min^−1^. Spectra were recorded in positive and negative modes at high resolution (30,000 FWHM (full width at half maximum)) and compared to authentic standards from the laboratory’s compound library including the three chlorogenic acid isomers: 3-caffeoylquinic acid, 4-caffeoylquinic acid and 5-caffeoylqunic acid.

Lyophilized nectar (original volume ~10 μL) was extracted in 50 μL methanol and injected directly onto an LC-MS system with a ZQ LC-MS detector on a Phenomenex Luna C18(2) column (150 × 4.0 mm i.d., 5 μm particle size) operating under gradient conditions, with A = MeOH, B = H_2_O, C = 1% HCO_2_H in MeCN; A = 0%, B = 90% at t = 0 min; A = 90%, B = 0% at t = 20 min; A = 90%, B = 0% at t = 30 min; A = 0%, B = 90% at t = 31 min; column temperature 30 °C and flow rate of 0.5 mL min^−1^. Aliquots (10 μL) were injected directly on to the column and components identified by comparison with pollen extracts analyzed as described above under HRESIMS. All chlorogenic acids were quantified against calibration curves of an authentic standard of 5-caffeoylquinic acid.

#### Identification of chlorogenic acids

All three chlorogenic acids have a molecular ion [M + H]^+^ with *m/z* = 355.1020 (calculated for C_16_H_19_O_9_^+^ = 355.1024) and a major diagnostic fragment *m/z* = 163.04 (calculated for C_9_H_7_O_3_^+^ = 163.039) from [M-quinic acid]^+^. The chlorogenic acids elute in the order 3-caffeoyl-, 5-caffeoyl- and 4-caffeoylquinic acids at 4.0 min, 5.6 min and 7.0 min respectively with the following diagnostic MS2 fragments in negative mode: 3-caffeoylquinic acid fragment *m/z* = 163, 4-caffeoylquinic acid fragment *m/z* = 173 and 5-caffeoylquinic acid fragment *m/z* = 191.

#### Statistical comparison between pollen and nectar

Within each of the three plant types for which we measured chlorogenic acids in both pollen and nectar—*M. domestica*, wild *V. corymbosum*, and cultivated *V. corymbosum*—we compared pollen and nectar 5-caffeoylquinic acid concentrations using an unpaired, two-sided Wilcoxon signed-rank test.

#### Analysis of thymol in *Thymus vulgaris* nectar

For analysis of thymol, dried nectar from a sample of known volume (~10 μL) was extracted in 250 μL of chloroform to which was added 500 ng of decyl acetate (50 μL of a 10 ng μL^−1^ solution) as an internal standard. The extract was injected directly onto an Agilent 6890 gas chromatograph coupled to an Agilent 5973 mass spectrometer with a DB-5 fused silica capillary column (30 m length, 0.25 mm diameter, 0.25 μm film thickness) (Agilent). The column temperature was held at 50 °C for 2 min, then heated to 240 °C at 6 °C min^−1^. The ion source was held at 150 °C, and the transfer line was held at 250 °C. Thymol was identified by comparison to a thymol standard (Sigma Ltd) and quantified using the fragment ion *m/z* = 135 relative to the Total Ion Chromatogram (TIC) for the decyl acetate internal standard. This ratio was corrected using a response factor, which was obtained by analyzing a standard sample containing equal concentrations of thymol and decyl acetate.

## Additional Information

**How to cite this article**: Palmer-Young, E. C. *et al*. Bumble bee parasite strains vary in resistance to phytochemicals. *Sci. Rep.* **6**, 37087; doi: 10.1038/srep37087 (2016).

**Publisher's note:** Springer Nature remains neutral with regard to jurisdictional claims in published maps and institutional affiliations.

## Supplementary Material

Supplementary Information

## Figures and Tables

**Table 1:** Comparison of phytochemical resistance in *Crithidia bombi*, other trypanosomes and parasites, animal cells, and insects.

Phytochemical	EC50 (ppm)	Species or cell type	Reference
**Anabasine**	**628–2160**	***Crithidia bombi***	**This study**
	>100	*Trypanosoma cruzi* (epimastigote)	81
	>100	*Spodoptera frugiperda* (Sf9) cells	81
	>100	CHO cells (hamster ovary)	81
	5–20	*Crithidia* (reduced infection in *Bombus impatiens)*	22
	20	*Crithidia* (reduced infection in *Bombus impatiens)*	82
	5	*Nectarinea osea* (sunbird feeding deterrent)	63
**Nicotine**	**>1000**	***Crithidia bombi***	**This study**
	>1000	*Trypanosoma brucei*	45
	2	*Crithidia* (reduced infection in *Bombus impatiens, B. terrestris*)	22,23
	2000	*Apis mellifera* (2 d LD50)	14
**Amygdalin**	**>10,000**	***Crithidia bombi***	**This study**
	>10,000	*Herpetomonas culicidarum* carbon source	83
	>2000	*Leishmania tropica*	84
	30	*Apis mellifera* (2 d LD50)	14
	2100	*Apis mellifera* (6 d LD50)	85
**Caffeic acid**	**>250**	***Crithidia bombi***	**This study**
	5.6	*Leishmania donovani* (amastigote)	24
	1.1	*Trypanosoma brucei rhodesiense* (bloodstream form)	24
	>30	*Trypanosoma cruzi* (trypomastigote)	24
	56	*Trypanosoma cruzi (trypomastigote)*	86
	53.3	L6 rat muscle cells	24
	109.1	Human lymphocytes	87
	>128	*Paenibacillus larvae* (American foulbrood–MIC)	88
	>300	*Culex quinquefasciatus* Say (mosquito) larvae	89
	>500 μg fly^−1^	*Musca domesticus* (housefly) adults	89
**Chlorogenic acid**^**#**^	**>2500**	***Crithidia bombi***	**This study**
	7	*Leishmania donovani* (unknown strain)	49
	>17.7	*Leishmania donovani* MHOMET- 67/L82	50
	18.9	*Trypanosoma brucei rhodesiense* (STIB 900)	49
	>10.6	*Trypanosoma brucei rhodesiense* (STIB 900)	50
	61	*Trypanosoma cruzi* (trypomastigote)	86
	>90	*Trypanosoma cruzi* (amastigote)	49
	>50	*Plasmodium falciparum*	49
	>3.5	*Plasmodium falciparum* K1 resistant strain	50
	>90	L6 rat muscle cells	49
	8149.13	Rat hepatocytes	90
	111.5	Human lymphocytes	87
	>12760	*Spodoptera eridania* larvae	91
**Eugenol**	**19.7–23.5**	***Crithidia bombi***	**This study**
	93.7	*Crithidia fasciculata*	27
	80	*Leishmania amazonensis*	92
	37.2	*Trypanosoma brucei brucei* TC221 (bloodstream form)	44
	246	*Trypanosoma cruzi*	27
	93	HL-60 (human leukemia)	44
	13	*Sarcoptes scabiei* mites (permethrin-sensitive)	93
	40	*Sarcoptes scabiei* mites (permethrin-resistant)	93
(clove oil*)	7800	*Apis mellifera* (8 d LD50)	70
(clove oil*)	240	*Apis mellifera* (14 d LD50)	70
**Gallic acid**	**>250**	***Crithidia bombi***	**This study**
	>30	*Leishmania donovani* (extracellular)	24
	>25.0	*Leishmania donovani* (extracellular)	26
	4.4	*Leishmania donovani* (intracellular)	26
	8.0	*Trypanosoma brucei brucei* (bloodstream form)	48
	5.1	*Trypanosoma brucei brucei* (procyclic form)	48
	1.6	*Trypanosoma brucei rhodesiense* (bloodstream form)	24
	67	*Trypanosoma cruzi*	24
	14.4	L6 rat muscle cells	24
	15.6	Mouse macrophages	26
	>300	*Culex quinquefasciatus* Say (mosquito) larvae	89
	>500 μg fly^−1^	*Musca domesticus* (housefly) adults	89
**β-caryophyllene**	**>0.050**	***Crithidia bombi***	**This study**
	13.78	*Trypanosoma brucei brucei* TC221 (bloodstream form)	44
	41.2	*Trypanosoma brucei brucei* Lister 427 (bloodstream form)	94
	>100	*Trypansoma brucei brucei* Lister 427 (procyclic form)	94
	0.002–0.004	*Pseudomonas syringae*	15
	221	*Heliothis virescens* (cell cultures)	95
	19.31	HL-60 (human leukemia)	44
	>300	*A. mellifera* (<300 ppm attractive)	14
**Thymol**	**4.53–22.2**	***Crithidia bombi***	**This study**
	32.5	*Crithidia fasciculata*	27
	22.9	*Trypanosoma brucei brucei*	44
	62	*Trypanosoma cruzi* (epimastigote)	25
	53	*Trypanosoma cruzi* (trypomastigote)	25
	64–128	*Paenibacillus larvae* (MIC)	88
	40.7	HL-60 (human leukemia)	44
	>1000	*Apis mellifera* (8 d LD50)	70
	30	*Culex quinquefasciatus* Say (mosquito) larvae	89
	53 μg fly^−1^	*Musca domesticus* (housefly) adults	89
(thyme oil)**	>10,000	*Apis mellifera* (2 d LD50)	14

Concentrations are from this study (**bold**) and the sources cited in the table. Values are in EC50 in ppm of pure compound unless otherwise noted. Within each compound, observations are arranged (if applicable) beginning with trypanosomes, then other pathogens, followed by animal cells and insects. Trypanosome EC50 values all refer to *in vitro* assays of cell cultures. See specific references for methodological details. ^#^Refers to 3-O-caffeoylquinic acid. *Clove (*Syzygium aromaticum*) oil: 86.7% eugenol^96^. **Thyme (*Thymus*) oil: 65.3% thymol^97^.

**Table 2:** Phytochemical concentrations in floral tissues, pollen, nectar, and honey.

Compound	Sample type	Plant species	Concentration (ppm)^*^	Reference
**Pyridine alkaloids**				
**Anabasine**				
	flowers	*N. noctiflora*	2351	40
	flowers	*N. petunioides*	1482	40
	nectar	*N. glauca*	5	63
	nectar	32 *Nicotiana* spp	0–1.52	40
	nectar	*N. tabacum*	0–1.0	98
**Nicotine**				
	nectar	32 *Nicotiana* spp.	0–5.38	40
	nectar	*N. attenuata*	4	41
	nectar	*N. glauca*	0.5	63
**Cyanogenic glycosides**				
**Amygdalin**				
	pollen	*Amygdalus communis*	1889	99
	nectar	*Amygdalus communis*	4–10	99
**Phenolics**				
***Hydroxycinnamic acids***				
**Caffeic acid**				
	honey	*Quercus robur*	26.8	53
	honey	*Tilia platyphyllos*	8.8	53
	honey	*Fagopyrum esculentum*	7.07	100
	honey	*Phlomis armeniaca*	6.6	53
	honey	*Eryngium campestre*	6.18	53
	honey	*Astragalus microcephalus*	5.14	53
	honey	*Castanea sativa*	4.83	53
**Chlorogenic acids**				
5-O-caffeoylquinic acid	pollen	*Persea americana*	1525 ± 486 SD (n = 30)	**This study**
5-O-caffeoylquinic acid	pollen	*Malus domestica*	475 ± 862 SD (n = 30)	**This study**
5-O-caffeoylquinic acid	pollen	*Vaccinium corymbosum* (cult.)	430 ± 404 SD (n = 53)	**This study**
5-O-caffeoylquinic acid	pollen	*Vaccinium corymbosum* (wild)	192 ± 204 SD (n = 30)	**This study**
3-O-caffeoylquinic acid	nectar	*Prunus dulcis*	25.0 ± 14.9 SD (n = 15)	**This study**
5-O-caffeoylquinic acid	nectar	*Malus domestica*	15.6 ± 15.2 SD (n = 30)	**This study**
5-O-caffeoylquinic acid	nectar	*Vaccinium corymbosum* (cult.)	14.6 ± 28.2 SD (n = 52)	**This study**
5-O-caffeoylquinic acid	nectar	*Vaccinium corymbosum* (wild)	7.52 ± 4.23 SD (n = 29)	**This study**
4-O-caffeoylquinic acid	nectar	*Vaccinium corymbosum* (wild)	6.66 ± 5.11 SD (n = 30)	**This study**
4-O-caffeoylquinic acid	nectar	*Vaccinium corymbosum* (cult.)	3.77 ± 7.62 SD (n = 55)	**This study**
3-O-caffeoylquinic acid	honey	*Leptospermum scoparium*	8.2	101
3-O-caffeoylquinic acid	honey	*Tilia* spp	0.21	100
3-O-caffeoylquinic acid	honey	*Brassica rapa*	0.17	100
***Phenylpropenes***				
**Eugenol**				
	bud essential oil	*Syzygium aromaticum*	86.70%	96
	floral essential oil	*Ocimum selloi*	66.20%	102
(methyl eugenol)	floral essential oil	*Rosa rugosa*	6.88%	103
	floral volatiles	*Rhizophora stylosa*	27.20%	104
	pollen volatiles	*Rosa rugosa*	>2%	73
(eugenol + methyl eugenol)	stamens	*Rosa x hybrida*	49.9	105
	petals (male)	*Cucurbita pepo* cv. Tosca	1.2	106
	petals (female)	*Cucurbita pepo* cv. Tosca	0.99	106
	anther	*Cucurbita pepo* cv. Tosca	0.57	106
	Nectar (male and female)	*Cucurbita pepo* cv. Tosca	trace	106
	stigma	*Cucurbita pepo* cv. Tosca	ND	106
	honey	*Rosmarinus* spp	0.02–0.03	107
	honey	*Thymus* spp	0.016	108
***Trihydroxybenzoic acids***				
**Gallic acid**				
	honey	*Quercus robur*	82.5	53
	honey	*Leptospermum scoparium*	70.5	101
	honey	*Leptospermum polygalifolium*	12.3	101
	honey	*Fagopyrum esculentum*	9.1	100
	honey	*Tilia* spp	3.26	100
	honey	*Brassica rapa*	1.27	100
	honey	*Castanea sativus*	0.91	53
	honey	*Calluna vulgaris*	0.61	53
**Terpenoids**				
**β-caryophyllene**				
	floral volatiles	*Arabidopsis thaliana*	40%	109
	floral volatiles	*Nicotiana sylvestris*	35%	110
	floral volatiles	*Dianthus caryophyllus*	23%	111
	floral volatiles	*Citrus limon*	9.50%	112
	pollen volatiles	*Citrus limon*	14.50%	112
	pollen volatiles	*Papaver rhoaeus*	>5%	113
	pollen volatiles	*Lupinus polyphyllus*	>5%	113
	pollen volatiles	*Laurus nobilis*	3.40%	114
	stamen volatiles	*Laurus nobilis*	15.40%	114
	flower bud volatiles	*Citrus limon*	11.90%	112
	petal volatiles	*Citrus limon*	2.50%	112
**Thymol**				
	nectar	*Thymus vulgaris* cv. Silver	8.2 (n = 1)	**This study**
	nectar	*Thymus vulgaris* cv. German	5.2 ± 2.98 SD (n = 11)	**This study**
	honey	Apigard™-treated hives	0.5–2.65	115
	honey	*Calluna vulgaris*	0.346	116
	honey	*Thymus* spp.	0.27	115
	honey	*Tilia* spp	0.16	117
	honey	*Erica* spp.)	0.142	116
	honey	*Erica* spp.	0.12	115

Concentration measurements for chlorogenic acid and thymol **(bold**) are from this study’s field sampling of nectar and pollen. Sample sizes are in parentheses. Concentrations of other phytochemicals were compiled through literature searches. Data are arranged in order of decreasing maximum concentration, first for sample types within compounds, and then by observations within a given sample type. SD: Standard Deviation. ^*^Units are mean concentration by mass in ppm, except for values followed by a “%” sign, which indicates % of total volatiles (for compounds where ppm concentrations were unavailable).

## References

[b1] McArt S. H., Koch H., Irwin R. E. & Adler L. S. Arranging the bouquet of disease: Floral traits and the transmission of plant and animal pathogens. Ecol. Lett. 17, 624–636 (2014). 24528408 10.1111/ele.12257

[b2] Vanbergen A. J. & Insect Pollinators Initiative. Threats to an ecosystem service: pressures on pollinators. Front. Ecol. Environ. 11, 251–259 (2013).

[b3] Goulson D., Nicholls E., Botías C. & Rotheray E. L. Bee declines driven by combined stress from parasites, pesticides, and lack of flowers. Science 347, 1255957 (2015). 25721506 10.1126/science.1255957

[b4] Cameron S. A. . Patterns of widespread decline in North American bumble bees. Proc. Natl. Acad. Sci. 108, 662–667 (2011). 21199943 10.1073/pnas.1014743108 PMC3021065

[b5] Singh R. . RNA viruses in hymenopteran pollinators: Evidence of inter-taxa virus transmission via pollen and potential impact on non-*Apis* Hymenopteran species. PLOS ONE 5, 1–16 (2010). 10.1371/journal.pone.0014357 PMC3008715 21203504

[b6] Fürst M. A., McMahon D. P., Osborne J. L., Paxton R. J. & Brown M. J. F. Disease associations between honeybees and bumblebees as a threat to wild pollinators. Nature 506, 364–366 (2014). 24553241 10.1038/nature12977 PMC3985068

[b7] Graystock P., Goulson D. & Hughes W. O. H. Parasites in bloom: flowers aid dispersal and transmission of pollinator parasites within and between bee species. Proc R Soc B 282, 20151371 (2015). 10.1098/rspb.2015.1371 PMC4632632 26246556

[b8] Arbetman M. P., Meeus I., Morales C. L., Aizen M. A. & Smagghe G. Alien parasite hitchhikes to Patagonia on invasive bumblebee. Biol. Invasions 15, 489–494 (2012).

[b9] Schmid-Hempel R. . The invasion of southern South America by imported bumblebees and associated parasites. J. Anim. Ecol. 83, 823–837 (2014). 24256429 10.1111/1365-2656.12185

[b10] Durrer S. & Schmid-Hempel P. Shared use of flowers leads to horizontal pathogen transmission. Proc. R. Soc. Lond. B Biol. Sci. 258, 299–302 (1994).

[b11] Heinrich B. Bumblebee Economics: Revised Edition. (Harvard University Press, 2004).

[b12] Adler L. S. The ecological significance of toxic nectar. Oikos 91, 409–420 (2001).

[b13] Dobson H. E. M. Survey of pollen and pollenkitt lipids—chemical cues to flower visitors? Am. J. Bot. 75, 170–182 (1988).

[b14] Detzel A. & Wink M. Attraction, deterrence or intoxication of bees (*Apis mellifera*) by plant allelochemicals. Chemoecology 4, 8–18 (1993).

[b15] Huang M. . The major volatile organic compound emitted from *Arabidopsis thaliana* flowers, the sesquiterpene *(E)*‐β‐caryophyllene, is a defense against a bacterial pathogen. New Phytol. 193, 997–1008 (2012). 22187939 10.1111/j.1469-8137.2011.04001.x

[b16] Karban R. & English-Loeb G. Tachinid parasitoids affect host plant choice by caterpillars to increase caterpillar survival. Ecology 78, 603–611 (1997).

[b17] Singer M., Mace K. & Bernays E. Self-medication as adaptive plasticity: increased ingestion of plant toxins by parasitized caterpillars. PLOS ONE 4, e4796 (2009). 19274098 10.1371/journal.pone.0004796 PMC2652102

[b18] Gowler C. D., Leon K. E., Hunter M. D. & Roode J. C. de. Secondary defense chemicals in milkweed reduce parasite infection in monarch butterflies, *Danaus plexippus*. J. Chem. Ecol. 41, 520–523 (2015). 25953502 10.1007/s10886-015-0586-6

[b19] Costa C., Lodesani M. & Maistrello L. Effect of thymol and resveratrol administered with candy or syrup on the development of *Nosema ceranae* and on the longevity of honeybees (*Apis mellifera* L.) in laboratory conditions. Apidologie 41, 141–150 (2010).

[b20] Gherman B. I. . Pathogen-associated self-medication behavior in the honeybee *Apis mellifera*. Behav. Ecol. Sociobiol. 68, 1777–1784 (2014).

[b21] Simone-Finstrom M. D. & Spivak M. Increased resin collection after parasite challenge: a case of self-medication in honey bees? PLOS ONE 7, e34601 (2012). 22479650 10.1371/journal.pone.0034601 PMC3315539

[b22] Richardson L. L. . Secondary metabolites in floral nectar reduce parasite infections in bumblebees. Proc. R. Soc. Lond. B Biol. Sci. 282 20142471 (2015). 10.1098/rspb.2014.2471 PMC4345440 25694627

[b23] Baracchi D., Brown M. J. F. & Chittka L. Behavioral evidence for self-medication in bumblebees? F1000Research 4, 1–15 (2015). 10.12688/f1000research.6262.1 PMC4406194 25949807

[b24] Tasdemir D. . Antitrypanosomal and antileishmanial activities of flavonoids and their analogues: *in vitro, in vivo*, structure-activity relationship, and quantitative structure-activity relationship studies. Antimicrob. Agents Chemother. 50, 1352–1364 (2006). 16569852 10.1128/AAC.50.4.1352-1364.2006 PMC1426963

[b25] Santoro G. F. . Effect of oregano (*Origanum vulgare* L.) and thyme (*Thymus vulgaris* L.) essential oils on *Trypanosoma cruzi* (Protozoa: Kinetoplastida) growth and ultrastructure. Parasitol. Res. 100, 783–790 (2007). 17024354 10.1007/s00436-006-0326-5

[b26] Kiderlen A. F., Kayser O., Ferreira D. & Kolodziej H. Tannins and related compounds: killing of amastigotes of *Leishmania donovani* and release of nitric oxide and tumour necrosis factor a in macrophages *in vitro*. Z. Für Naturforschung C 56, 444–454 (2001). 10.1515/znc-2001-5-620 11421463

[b27] Azeredo C. M. O. & Soares M. J. Combination of the essential oil constituents citral, eugenol and thymol enhance their inhibitory effect on *Crithidia fasciculata* and *Trypanosoma cruzi* growth. Rev. Bras. Farmacogn. 23, 762–768 (2013).

[b28] Lipa J. & Triggiani O. *Crithidia bombi* sp. n. a flagellated parasite of a bumble-bee *Bombus terrestris* L. (Hymenoptera, Apidae). Acta Protozool. 27, 287–290 (1988).

[b29] Yourth C. P., Brown M. J. F. & Schmid-Hempel P. Effects of natal and novel *Crithidia bombi* (Trypanosomatidae) infections on *Bombus terrestris* hosts. Insectes Sociaux 55, 86–90 (2007).

[b30] Fauser-Misslin A., Sadd B. M., Neumann P. & Sandrock C. Influence of combined pesticide and parasite exposure on bumblebee colony traits in the laboratory. J. Appl. Ecol. 51, 450–459 (2014).

[b31] Brown M. J. F., Schmid‐Hempel R. & Schmid‐Hempel P. Strong context‐dependent virulence in a host–parasite system: reconciling genetic evidence with theory. J. Anim. Ecol. 72, 994–1002 (2003).

[b32] Sadd B. M. & Barribeau S. M. Heterogeneity in infection outcome: lessons from a bumblebee-trypanosome system. Parasite Immunol. 35, 339–349 (2013). 23758554 10.1111/pim.12043

[b33] Manson J. S., Otterstatter M. C. & Thomson J. D. Consumption of a nectar alkaloid reduces pathogen load in bumble bees. Oecologia 162, 81–89 (2010). 19711104 10.1007/s00442-009-1431-9

[b34] Cisarovsky G. & Schmid-Hempel P. Combining laboratory and field approaches to investigate the importance of flower nectar in the horizontal transmission of a bumblebee parasite. Entomol. Exp. Appl. 152, 209–215 (2014).

[b35] Biller O. M., Adler L. S., Irwin R. E., McAllister C. & Palmer-Young E. C. Possible synergistic effects of thymol and nicotine against *Crithidia bombi* parasitism in bumble bees. PLoS ONE 10, e0144668 (2015). 26657643 10.1371/journal.pone.0144668 PMC4686078

[b36] Thorburn L. P., Adler L. S., Irwin R. E. & Palmer-Young E. C. Variable effects of nicotine, anabasine, and their interactions on parasitized bumble bees. F1000Research 4, 880 (2015). 26998225 10.12688/f1000research.6870.1 PMC4786900

[b37] Erler S., Popp M., Wolf S. & Lattorff H. M. G. Sex, horizontal transmission, and multiple hosts prevent local adaptation of *Crithidia bombi*, a parasite of bumblebees (*Bombus* spp.). Ecol. Evol. 2, 930–940 (2012). 22837838 10.1002/ece3.250 PMC3399159

[b38] Friedrich M. J. Artemisinin-resistant malaria. JAMA 307, 2017–2017 (2012).

[b39] Dušan F., Marián S., Katarína D. & Dobroslava B. Essential oils-their antimicrobial activity against *Escherichia coli* and effect on intestinal cell viability. Toxicol. In Vitro 20, 1435–1445 (2006). 16919909 10.1016/j.tiv.2006.06.012

[b40] Adler L. S., Seifert M. G., Wink M. & Morse G. E. Reliance on pollinators predicts defensive chemistry across tobacco species. Ecol. Lett. 15, 1140–1148 (2012). 22834564 10.1111/j.1461-0248.2012.01838.x

[b41] Kessler D. . Unpredictability of nectar nicotine promotes outcrossing by hummingbirds in *Nicotiana attenuata*. Plant J. 71, 529–538 (2012). 22448647 10.1111/j.1365-313X.2012.05008.x

[b42] Egan P. A. . Plant toxin levels in nectar vary spatially across native and introduced populations. J. Ecol. 104, 1106–1115 (2016).

[b43] Santoro G. F., Cardoso M. G., Guimarães L. G. L., Mendonça L. Z. & Soares M. J. *Trypanosoma cruzi*: activity of essential oils from *Achillea millefolium* L., *Syzygium aromaticum* L. and *Ocimum basilicum* L. on epimastigotes and trypomastigotes. Exp. Parasitol. 116, 283–290 (2007). 17349626 10.1016/j.exppara.2007.01.018

[b44] Nibret E. & Wink M. Trypanocidal and antileukaemic effects of the essential oils of *Hagenia abyssinica, Leonotis ocymifolia, Moringa stenopetala*, and their main individual constituents. Phytomedicine 17, 911–920 (2010). 20359874 10.1016/j.phymed.2010.02.009

[b45] Merschjohann K., Sporer F., Steverding D. & Wink M. *In vitro* effect of alkaloids on bloodstream forms of *Trypanosoma brucei* and *T. congolense*. Planta Med. 67, 623–627 (2001). 11582539 10.1055/s-2001-17351

[b46] Schmid-Hempel R. & Tognazzo M. Molecular divergence defines two distinct lineages of *Crithidia bombi* (Trypanosomatidae), parasites of bumblebees. J. Eukaryot. Microbiol. 57, 337–345 (2010). 20497286 10.1111/j.1550-7408.2010.00480.x

[b47] Salathé R., Tognazzo M., Schmid-Hempel R. & Schmid-Hempel P. Probing mixed-genotype infections I: Extraction and cloning of infections from hosts of the trypanosomatid *Crithidia bombi*. PLOS ONE 7, e49046 (2012). 23155449 10.1371/journal.pone.0049046 PMC3498296

[b48] Koide T. . Trypanocidal effects of gallic acid and related compounds. Planta Med. 64, 27–30 (1998). 9491765 10.1055/s-2006-957360

[b49] Kırmızıbekmez H. . Inhibiting activities of the secondary metabolites of *Phlomis brunneogaleata* against parasitic protozoa and plasmodial enoyl-ACP reductase, a crucial enzyme in fatty acid biosynthesis. Planta Med. 70, 711–717 (2004). 15326547 10.1055/s-2004-827200

[b50] Lagnika L., Weniger B., Senecheau C. & Sanni A. Antiprotozoal activities of compounds isolated from *Croton lobatus* L. *Afr*. J. Infect. Dis. 3, 1–5 (2010).

[b51] Baker H. G. Non-sugar chemical constituents of nectar. Apidologie 8, 349–356 (1977).

[b52] Hurst V., Stevenson P. C. & Wright G. A. Toxins induce ‘malaise’ behaviour in the honeybee (*Apis mellifera*). J. Comp. Physiol. A 200, 881–890 (2014). 10.1007/s00359-014-0932-0 PMC4169619 25149875

[b53] Can Z. . An investigation of Turkish honeys: Their physico-chemical properties, antioxidant capacities and phenolic profiles. Food Chem. 180, 133–141 (2015). 25766810 10.1016/j.foodchem.2015.02.024

[b54] Patthamakanokporn O., Puwastien P., Nitithamyong A. & Sirichakwal P. P. Changes of antioxidant activity and total phenolic compounds during storage of selected fruits. J. Food Compos. Anal. 21, 241–248 (2008).

[b55] Leja M. . Antioxidative properties of bee pollen in selected plant species. Food Chem. 100, 237–240 (2007).

[b56] Rahnamaeian M. . Insect antimicrobial peptides show potentiating functional interactions against Gram-negative bacteria. Proc R Soc B 282, 20150293 (2015). 10.1098/rspb.2015.0293 PMC4426631 25833860

[b57] Cariveau D. P., Elijah Powell J., Koch H., Winfree R.& Moran N. A. Variation in gut microbial communities and its association with pathogen infection in wild bumble bees (*Bombus*). ISME J. 8, 2369–2379 (2014). 24763369 10.1038/ismej.2014.68 PMC4260702

[b58] Maggi M. . Effects of the organic acids produced by a lactic acid bacterium in *Apis mellifera* colony development, *Nosema ceranae* control and fumagillin efficiency. Vet. Microbiol. 167, 474–483 (2013). 23978352 10.1016/j.vetmic.2013.07.030

[b59] Schnürer J. & Magnusson J. Antifungal lactic acid bacteria as biopreservatives. Trends Food Sci. Technol. 16, 70–78 (2005).

[b60] Borchers A. T., Hackman R. M., Keen C. L., Stern J. S. & Gershwin M. E. Complementary medicine: a review of immunomodulatory effects of Chinese herbal medicines. Am. J. Clin. Nutr. 66, 1303–1312 (1997). 9394679 10.1093/ajcn/66.6.1303

[b61] Mao W., Schuler M. A. & Berenbaum M. R. Honey constituents up-regulate detoxification and immunity genes in the western honey bee *Apis mellifera*. Proc. Natl. Acad. Sci. USA 110, 8842–8846 (2013). 23630255 10.1073/pnas.1303884110 PMC3670375

[b62] Fujisawa S., Atsumi T., Kadoma Y. & Sakagami H. Antioxidant and prooxidant action of eugenol-related compounds and their cytotoxicity. Toxicology 177, 39–54 (2002). 12126794 10.1016/s0300-483x(02)00194-4

[b63] Tadmor-Melamed H. . Limited ability of Palestine sunbirds *Nectarinia osea* to cope with pyridine alkaloids in nectar of tree tobacco *Nicotiana glauca*. Funct. Ecol. 18, 844–850 (2004).

[b64] Kwong W. K. & Moran N. A. Cultivation and characterization of the gut symbionts of honey bees and bumble bees: description of *Snodgrassella alvi* gen. nov., sp. nov., a member of the family Neisseriaceae of the Betaproteobacteria, and *Gilliamella apicola* gen. nov., sp. nov., a member of Orbaceae fam. nov., Orbales ord. nov., a sister taxon to the order ‘Enterobacteriales’ of the Gammaproteobacteria. Int. J. Syst. Evol. Microbiol. 63, 2008–2018 (2013). 23041637 10.1099/ijs.0.044875-0

[b65] Giacomelli A. . Combination of thymol treatment (Apiguard®) and caging the queen technique to fight *Varroa destructor*. Apidologie 1–11, doi: 10.1007/s13592-015-0408-4 (2015).

[b66] Heinrich B. The foraging specializations of individual bumblebees. Ecol. Monogr. 46, 105–128 (1976).

[b67] Svircev A. M. . Effects of thymol fumigation on survival and ultrastracture of *Monilinia fructicola*. Postharvest Biol. Technol. 45, 228–233 (2007).

[b68] Pina-Vaz C. . Antifungal activity of *Thymus* oils and their major compounds. J. Eur. Acad. Dermatol. Venereol. 18, 73–78 (2004). 14678536 10.1111/j.1468-3083.2004.00886.x

[b69] Bakkali F., Averbeck S., Averbeck D. & Idaomar M. Biological effects of essential oils – A review. Food Chem. Toxicol. 46, 446–475 (2008). 17996351 10.1016/j.fct.2007.09.106

[b70] Ebert T. A., Kevan P. G., Bishop B. L., Kevan S. D. & Downer R. A. Oral toxicity of essential oils and organic acids fed to honey bees (*Apis mellifera*). J. Apic. Res. 46, 220–224 (2007).

[b71] Dobson H. E., Danielson E. M. & Wesep I. D. V. Pollen odor chemicals as modulators of bumble bee foraging on *Rosa rugosa* Thunb. (Rosaceae). Plant Species Biol. 14, 153–166 (1999).

[b72] Tan K. H. & Nishida R. Methyl eugenol: its occurrence, distribution, and role in nature, especially in relation to insect behavior and pollination. J. Insect Sci. 12, 1–74 (2012). 10.1673/031.012.5601 PMC3500151 22963669

[b73] Dobson H. E. M. & Bergstrom G. The ecology and evolution of pollen odors. Plant Syst. Evol. 222, 63–87 (2000).

[b74] Goyret J. & Farina W. M. Non-random nectar unloading interactions between foragers and their receivers in the honeybee hive. Naturwissenschaften 92, 440–443 (2005). 16133104 10.1007/s00114-005-0016-7

[b75] Marxer M., Vollenweider V. & Schmid-Hempel P. Insect antimicrobial peptides act synergistically to inhibit a trypanosome parasite. Phil Trans R Soc B 371, 20150302 (2016). 27160603 10.1098/rstb.2015.0302 PMC4874398

[b76] Core Team R. R: A language and environment for statistical computing. (2014).

[b77] Kahm M., Hasenbrink G., Lichtenberg-Fraté H., Ludwig J. & Kschischo M. grofit: fitting biological growth curves with R. J. Stat. Softw. 33, 1–21 (2010).20808728

[b78] Plummer M. JAGS: A program for analysis of Bayesian graphical models using Gibbs sampling. In Proceedings of the 3rd International Workshop on Distributed Statistical Computing (DSC 2003), https://www.r-project.org/conferences/DSC-2003/Drafts/Plummer.pdf (2003).

[b79] Plummer M. rjags: Bayesian graphical models using MCMC. CRAN Repos., https://CRAN.R-project.org/package=rjags (2016).

[b80] Arnold S. E. J., Idrovo M. E. P., Arias L. J. L., Belmain S. R. & Stevenson P. C. Herbivore defence compounds occur in pollen and reduce bumblebee colony fitness. J. Chem. Ecol. 40, 878–881 (2014). 24952086 10.1007/s10886-014-0467-4

[b81] González-Coloma A. . Structural diversity and defensive properties of norditerpenoid alkaloids. J. Chem. Ecol. 30, 1393–1408 (2004). 15503527 10.1023/b:joec.0000037747.74665.0a

[b82] Anthony W. E., Palmer-Young E. C., Leonard A. S., Irwin R. E. & Adler L. S. Testing dose-dependent effects of the nectar alkaloid anabasine on trypanosome parasite loads in adult bumble bees. PLOS ONE 10, e0142496 (2015). 26545106 10.1371/journal.pone.0142496 PMC4636389

[b83] Noguchi H. Comparative studies of Herpetomonads and Leishmanias II. Differentiation of the organisms by serological reactions and fermentation tests. J. Exp. Med. 44, 327–337 (1926). 19869187 10.1084/jem.44.3.327 PMC2131776

[b84] Dubois A. Utilization of sugars by *Leishmania trópica*. C. r. Seances Soc. Biol. 123, 141–144 (1936).

[b85] Kevan P. G. & Ebert T. Can almond nectar & pollen poison honey bees? Am. Bee J. June, 507–509 (2005).

[b86] Grecco S. S. . Anti-trypanosomal phenolic derivatives from *Baccharis uncinella*. Nat. Prod. Commun. 9, 171–173 (2014). 24689283

[b87] Cherng J.-M., Shieh D.-E., Chiang W., Chiang M.-Y. & Chiang L.-C. Chemopreventive effects of minor dietary constituents in common foods on human cancer cells. Biosci. Biotechnol. Biochem. 71, 1500–1504 (2007). 17587681 10.1271/bbb.70008

[b88] Flesar J. . *In vitro* growth-inhibitory effect of plant-derived extracts and compounds against *Paenibacillus larvae* and their acute oral toxicity to adult honey bees. Vet. Microbiol. 145, 129–133 (2010). 20409652 10.1016/j.vetmic.2010.03.018

[b89] Pavela R. Insecticidal properties of phenols on *Culex quinquefasciatus* Say and *Musca domestica* L. Parasitol. Res. 109, 1547–1553 (2011). 21523422 10.1007/s00436-011-2395-3

[b90] Moridani M. Y., Scobie H. & O’Brien P. J. Metabolism of caffeic acid by isolated rat hepatocytes and subcellular fractions. Toxicol. Lett. 133, 141–151 (2002). 12119122 10.1016/s0378-4274(02)00105-4

[b91] Lindroth R. L. & Peterson S. S. Effects of plant phenols on performance of southern armyworm larvae. Oecologia 75, 189, 185 (1988). 10.1007/BF00378595 28310832

[b92] Ueda-Nakamura T. . Antileishmanial activity of eugenol-rich essential oil from *Ocimum gratissimum*. Parasitol. Int. 55, 99–105 (2006). 16343984 10.1016/j.parint.2005.10.006

[b93] Pasay C. . Acaricidal activity of eugenol based compounds against scabies mites. PLOS ONE 5, e12079 (2010). 20711455 10.1371/journal.pone.0012079 PMC2920318

[b94] Bero J. . Antitrypanosomal compounds from the essential oil and extracts of *Keetia leucantha* leaves with inhibitor activity on *Trypanosoma brucei* glyceraldehyde-3-phosphate dehydrogenase. Phytomedicine 20, 270–274 (2013). 23312849 10.1016/j.phymed.2012.10.010

[b95] Stipanovic R. D., Elissalde M. H., Altman D. W. & Norman J. O. Cell culture bioassay to evaluate allelochemical toxicity to *Heliothis virescens* (Lepidoptera: Noctuidae). J. Econ. Entomol. 83, 737–741 (1990).

[b96] Maggi M. D. . Laboratory evaluations of *Syzygium aromaticum* (L.) Merr. et Perry essential oil against *Varroa destructor*. J. Essent. Oil Res. 22, 119–122 (2010).

[b97] Damiani N., Gende L. B., Bailac P., Marcangeli J. A. & Eguaras M. J. Acaricidal and insecticidal activity of essential oils on *Varroa destructor* (Acari: Varroidae) and *Apis mellifera* (Hymenoptera: Apidae). Parasitol. Res. 106, 145–152 (2009). 19795133 10.1007/s00436-009-1639-y

[b98] Adler L. S., Wink M., Distl M. & Lentz A. J. Leaf herbivory and nutrients increase nectar alkaloids. Ecol. Lett. 9, 960–967 (2006). 16913940 10.1111/j.1461-0248.2006.00944.x

[b99] London-Shafir I., Shafir S. & Eisikowitch D. Amygdalin in almond nectar and pollen – facts and possible roles. Plant Syst. Evol. 238, 87–95 (2003).

[b100] Socha R. . Phenolic profile and antioxidant properties of Polish honeys. Int. J. Food Sci. Technol. 46, 528–534 (2011).

[b101] Yao L. . Flavonoids, phenolic acids and abscisic acid in Australian and New Zealand *Leptospermum* honeys. Food Chem. 81, 159–168 (2003).

[b102] Martins E. R., Casali V. W. D., Barbosa L. C. A. & Carazza F. Essential oil in the taxonomy of *Ocimum selloi* benth. J. Braz. Chem. Soc. 8, 29–32 (1997).

[b103] Wu C. . The main chemical components of the essential oil from *Rosa rugosa* Thunb. Acta Bot. Sin. 27, 510–515 (1985).

[b104] Azuma H., Toyota M., Asakawa Y., Takaso T. & Tobe H. Floral scent chemistry of mangrove plants. J. Plant Res. 115, 0047–0053 (2002). 10.1007/s102650200007 12884048

[b105] Bergougnoux V. . Both the adaxial and abaxial epidermal layers of the rose petal emit volatile scent compounds. Planta 226, 853–866 (2007). 17520281 10.1007/s00425-007-0531-1

[b106] Granero A. M., Gonzalez F. J. E., Sanz J. M. G. & Vidal J. L. M. Analysis of biogenic volatile organic compounds in zucchini flowers: identification of scent sources. J. Chem. Ecol. 31, 2309–2322 (2005). 16195845 10.1007/s10886-005-7103-2

[b107] Castro-Vázquez L., Pérez-Coello M. S. & Cabezudo M. D. Analysis of volatile compounds of rosemary honey. Comparison of different extraction techniques. Chromatographia 57, 227–233 (2003).

[b108] Alissandrakis E., Tarantilis P. A., Pappas C., Harizanis P. C. & Polissiou M. Ultrasound-assisted extraction gas chromatography–mass spectrometry analysis of volatile compounds in unifloral thyme honey from Greece. Eur. Food Res. Technol. 229, 365–373 (2009).

[b109] Chen F. . Biosynthesis and emission of terpenoid volatiles from *Arabidopsis* flowers. Plant Cell 15, 481–494 (2003). 12566586 10.1105/tpc.007989 PMC141215

[b110] Loughrin J. H., Hamilton-Kemp T. R., Andersen R. A. & Hildebrand D. F. Headspace compounds from flowers of *Nicotiana tabacum* and related species. J. Agric. Food Chem. 38, 455–460 (1990).

[b111] Lavy M. . Linalool and linalool oxide production in transgenic carnation flowers expressing the *Clarkia breweri* linalool synthase gene. Mol. Breed. 9, 103–111 (2002).

[b112] Flamini G., Tebano M. & Cioni P. L. Volatiles emission patterns of different plant organs and pollen of *Citrus limon*. Anal. Chim. Acta 589, 120–124 (2007). 17397661 10.1016/j.aca.2007.02.053

[b113] Dobson H. E. M., Groth I. & Bergstrom G. Pollen advertisement: chemical contrasts between whole-flower and pollen odors. Am. J. Bot. 83, 877–885 (1996).

[b114] Flamini G., Cioni P. L. & Morelli I. Differences in the fragrances of pollen and different floral parts of male and female flowers of *Laurus nobilis*. J. Agric. Food Chem. 50, 4647–4652 (2002). 12137491 10.1021/jf020269x

[b115] Nozal M. J., Bernal J. L., Jiménez J. J., González M. J. & Higes M. Extraction of thymol, eucalyptol, menthol, and camphor residues from honey and beeswax: Determination by gas chromatography with flame ionization detection. J. Chromatogr. A 954, 207–215 (2002). 12058905 10.1016/s0021-9673(02)00153-x

[b116] Viñas P., Soler-Romera M. J. & Hernández-Córdoba M. Liquid chromatographic determination of phenol, thymol and carvacrol in honey using fluorimetric detection. Talanta 69, 1063–1067 (2006). 18970682 10.1016/j.talanta.2005.12.030

[b117] Guyot C., Bouseta A., Scheirman V. & Collin S. Floral origin markers of chestnut and lime tree honeys. J. Agric. Food Chem. 46, 625–633 (1998). 10554289 10.1021/jf970510l

