# Supplementary material for: Bumble bee parasite strains vary in resistance to phytochemicals
Source: Sci Rep. 2016 Nov 24;6:37087. doi: 10.1038/srep37087 (PMC5121629; doi:10.1038/srep37087)
Supplement: Supplementary Information [file srep37087-s1.pdf]

Supplementary Information

Bumble bee parasite strains vary in resistance to phytochemicals

Evan C. Palmer-Young, Ben M. Sadd, Philip C. Stevenson, Rebecca E. Irwin, Lynn S. Adler

Supplementary Table S1. Details of field sampling. Species and cultivars, locations, and dates of sampling.

Species	Site	Site GPS	Cultivars	Inclusive dates of sampling
Persea americana	South Coast Research and Extension Center (CA)	33°41'39.38"N 117°43'15.27"W	BL516/Marvel, Hass, and 46-4918-99	27 Feb - 4 March 2015
Malus domestica	Cold Spring Orchard	42°15'6.73"N 72°21'38.99"W	Macintosh, Honeycrisp, Fuji-Autumn Rose	13 - 18 May 2014
Prunus dulcis	Long Almond Orchard, Zamora, CA	38°49'25.88"N, 121°55'35.22"W	Nonpareil, Monterey and Winters	18 Feb 2015
Vaccinium corymbosum (cult.)	Cold Spring Orchard	42°15'6.73"N 72°21'38.99"W	Liberty, Patriot, Reka, Northland, Friendship, Bonus	19-27 May 2014
	Kenburn Orchard	42°36'43.44"N 72°39'18.05"W	Patriot	20-21 May 2014
	Sobieski's River Valley Farm	42°27'12.72"N 72°35'43.02"W	Spartan, Bluecrop	27 May 2014
	Nourse Farm	42°25'49.45"N 72°35'18.92"W	Spartan, Bluecrop	28 May 2014
Vaccinium corymbosum (wild)	Quabbin Reservoir Gate 10	42°23.537'N 72°24.181'W	NA	22 May 2014
	Harvard Pond	42°29'54.93"N, 72°12'47.83"W	NA	23-25 May 2014
	Harvard Forest	42°32'6.41"N, 72°11'19.53"W	NA	26 May 2014
Thymus vulgaris	S. Deerfield Farm	42°28'45.53"N 72°34'46.06"W	Silver	7 July 2015
Thymus vulgaris	S. Deerfield Farm	42°28'45.53"N 72°34'46.06"W	German	12 June - 7 July 2015

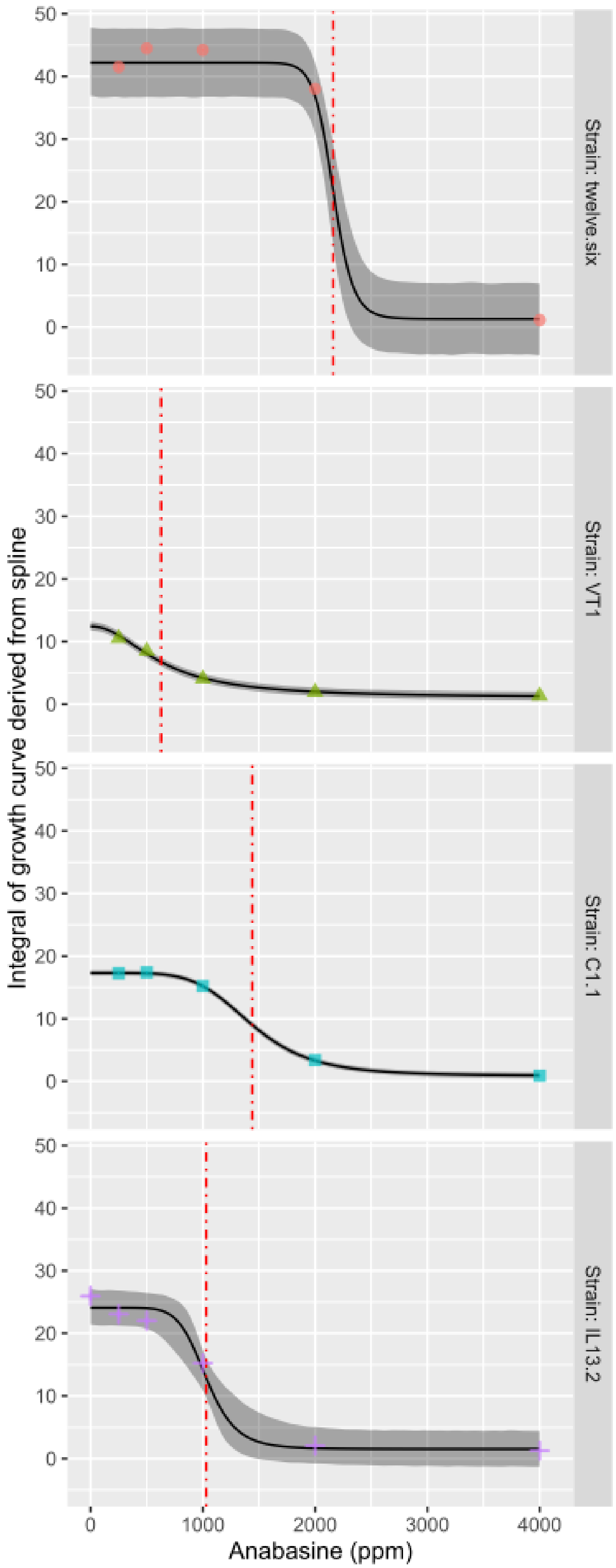

Supplementary Figure S1: 95% posterior density bands for responses of 4 parasite strains to anabasine. Points show raw data (mean +/- SD). Vertical lines indicate EC50 values.

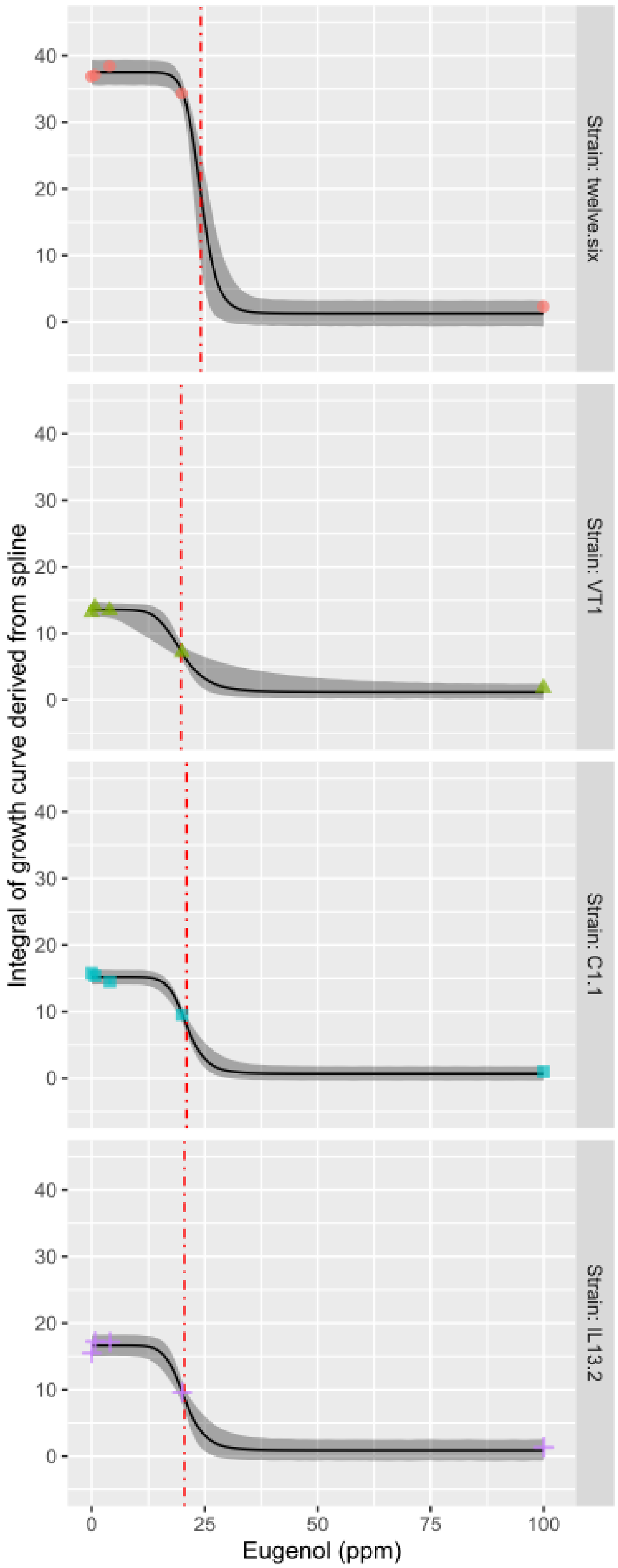

Supplementary Figure S2: 95% posterior density bands for responses of 4 parasite strains to eugenol. Points show raw data (mean +/- SD). Vertical lines indicate EC50 values.

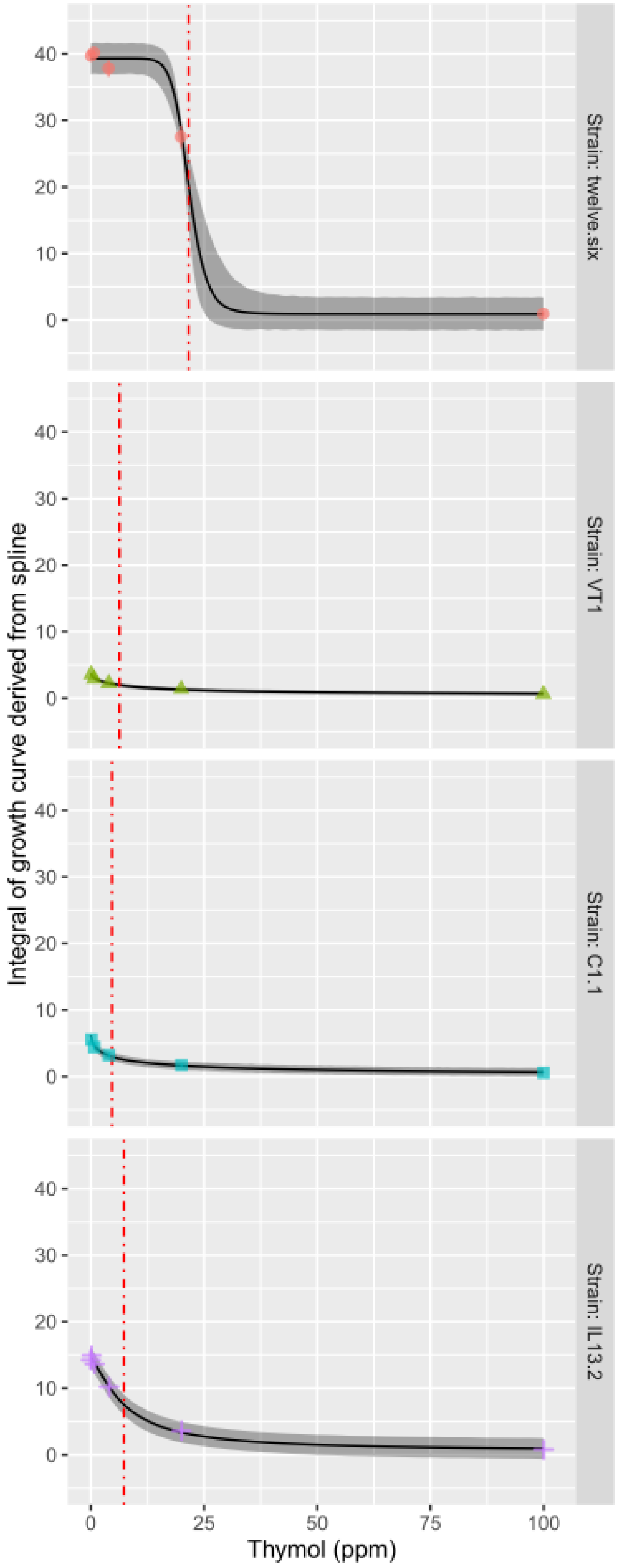

Supplementary Figure S3: 95% posterior density bands for responses of 4 parasite strains to thymol. Points show raw data (mean +/- SD). Vertical lines indicate EC50 values.

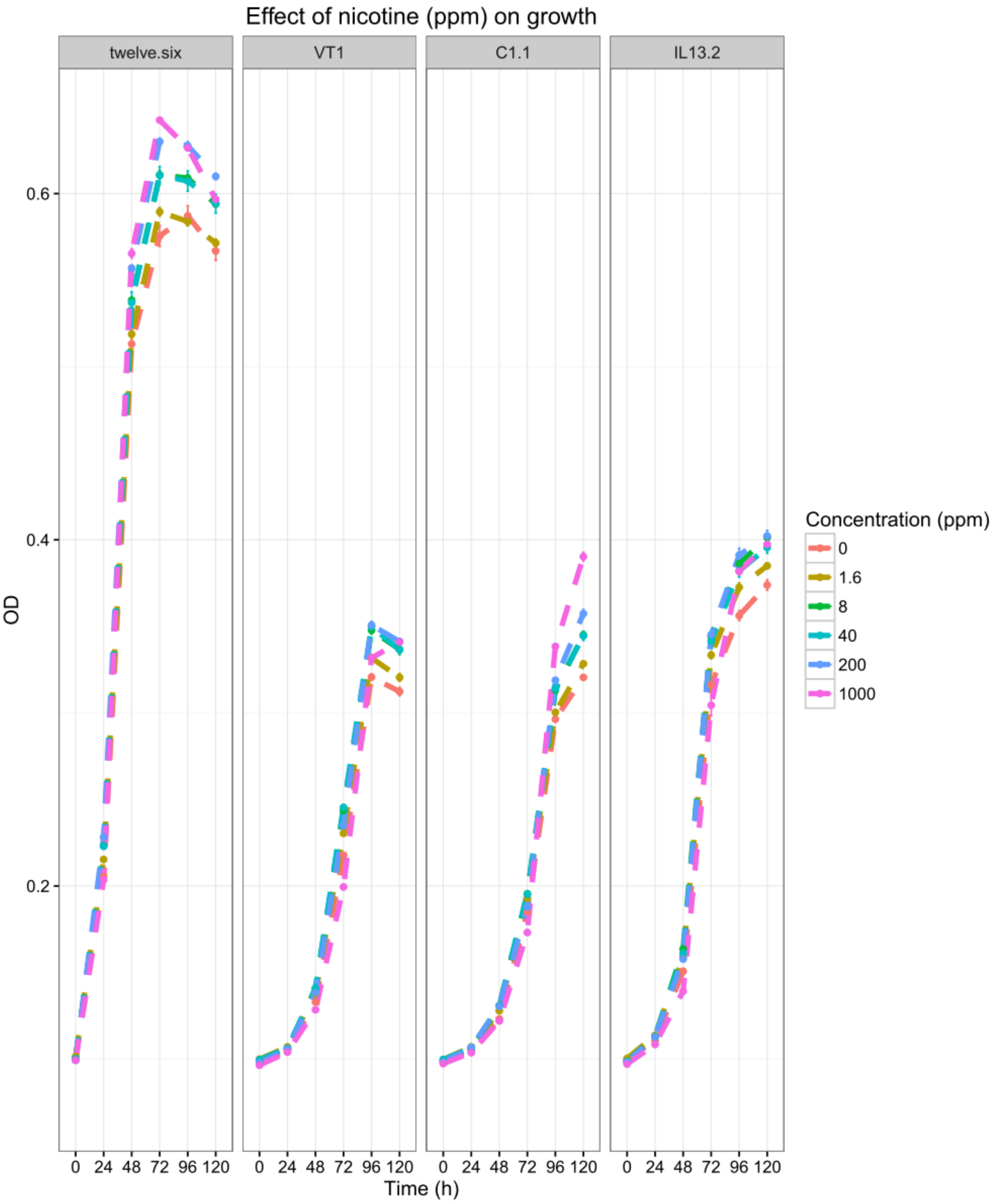

Supplementary Figure S4. Representative growth curves for four *C. bombi* strains in presence of non-inhibitory chemical nicotine. X axis shows time of measurement, Y axis shows optical density (OD 630 nm), points show mean +/- SD of 8 replicate wells. Lines are colored according to nicotine concentration.
